# Supplementary material for: Diagnostic Performance of Xpert MTB/RIF Ultra Compared with Predecessor Test, Xpert MTB/RIF, in a Low TB Incidence Setting: a Retrospective Service Evaluation
Source: Microbiol Spectr. 2022 Apr 26;10(3):e02345-21. doi: 10.1128/spectrum.02345-21 (PMC9241712; doi:10.1128/spectrum.02345-21)
Supplement: SUPPLEMENTAL FILE 1 — Supplemental material. Download spectrum.02345-21-s001.pdf, PDF file, 0.1 MB [file spectrum.02345-21-s001.pdf]

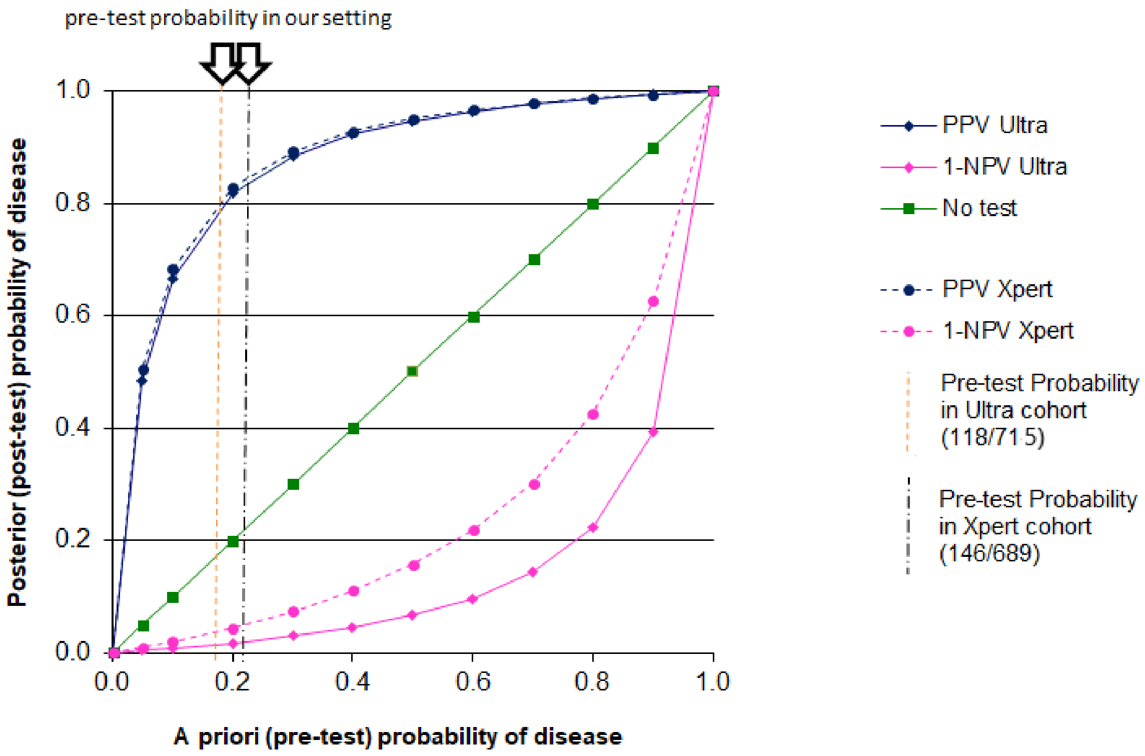

Supplementary Figure 1. Post-test Probability of TB based on Sensitivities and Specificities of Xpert and Ultra Assays. *Xpert: Xpert MTB/RIF, Ultra:* Xpert MTB/RIF Ultra, PPV: Positive Predictive Value, NPV: Negative Predictive Value. Graph created using Bayes Graph Creator (1). Blue lines indicate PPV or proportion of true positive results for each assay. Pink lines indicate proportion of false negative results for each assay, 1 -NPV.

Supplementary Table 1. Comparison of Ultra (and Xpert) Group Semiquantitative Result versus Smear Microscopy Result

Ultra (Xpert) semi-quantitative result	Smear Microscopy						Total	% Smear positive
	Negative	Scanty positive	1+	2+	3+	Smear positive *		
**High**	0 (0)	0 (0)	2 (0)	3 (3)	29 (29)	4 (-)	38 (32)	100 (100)
**Medium**	1 (0)	3 (1)	2 (4)	8 (7)	8 (18)	2 (-)	24(30)	95.8 (100)
**Low**	9 (3)	6 (14)	6 (8)	4 (6)	0 (1)	1 (-)	26 (32)	65.4 (90.6)
**Very Low**	7 (10)	7 (16)	2 (0)	0(0)	0 (0)	0 (-)	16 (26)	56.3 (61.5)
**Trace**	6 (-)	0 (-)	0 (-)	0 (-)	0 (-)	0 (-)	6 (-)	0 (-)
**Negative**	8 (23)	0 (3)	0 (0)	0 (0)	0 (0)	0 (-)	8 (26)	0 (11.5)
**Total**	31 (36)	16 (34)	12 (12)	15 (16)	37 (48)	7(-)	118 (146)	73.7 (75.3)

*Results in parenthesis correspond to specimens from Xpert cohort. 1+,2+,3+ correspond to AFB smear grade. AFB smear grades were designated as per the HPSC Guidelines 2010 for Grading Auramine smears (2). *7 of the 87 smear and culture positive samples from the Ultra cohort did not have smear grade result recorded and so are included in a separate column.*

References:

1. Arend SM, van Soolingen D. Performance of Xpert MTB/RIF Ultra: a matter of dead or alive. The Lancet Infectious diseases. 2018;18(1):8-10.
2. Health Protection Surveillance Centre. Guidelines on the Prevention and Control of Tuberculosis in Ireland 2010.
